# Melanin and Melanin-Functionalized Nanoparticles as Promising Tools in Cancer Research—A Review

**DOI:** 10.3390/cancers14071838

**Published:** 2022-04-06

**Authors:** Iasmina Marcovici, Dorina Coricovac, Iulia Pinzaru, Ioana Gabriela Macasoi, Roxana Popescu, Raul Chioibas, Istvan Zupko, Cristina Adriana Dehelean

**Affiliations:** 1 Faculty of Pharmacy, “Victor Babes” University of Medicine and Pharmacy Timisoara, Eftimie Murgu Square No. 2, 300041 Timisoara, Romania; iasmina.marcovici@umft.ro (I.M.); dorinacoricovac@umft.ro (D.C.); macasoi.ioana@umft.ro (I.G.M.); cadehelean@umft.ro (C.A.D.); 2 Research Center for Pharmaco-Toxicological Evaluations, Faculty of Pharmacy, “Victor Babes” University of Medicine and Pharmacy Timisoara, Eftimie Murgu Square No. 2, 300041 Timisoara, Romania; 3 Faculty of Medicine, “Victor Babes” University of Medicine and Pharmacy Timisoara, Eftimie Murgu Square No. 2, 300041 Timisoara, Romania; popescu.roxana@umft.ro (R.P.); office@medcom.ro (R.C.); 4 Research Center ANAPATMOL, Faculty of Medicine, “Victor Babes” University of Medicine and Pharmacy Timisoara, Eftimie Murgu Square No. 2, 300041 Timisoara, Romania; 5 Faculty of Pharmacy, University of Szeged, Eötvös u. 6, H-6720 Szeged, Hungary; zupko.istvan@szte.hu

**Keywords:** cancer, melanin, polydopamine, nanotechnology, nanoparticles, targeted delivery

## Abstract

**Simple Summary:**

Although a notable evolution was recorded in the field of cancer, both in terms of therapeutic options and diagnostic tools, with nanotechnology contributing significantly to this direction, cancer remains one of the leading causes of death globally. In recent years, the research community has proposed novel therapeutic approaches showing promising results, such as adjoining natural compounds possessing anticancer activity and nanotechnology. A natural compound that proved to have great potential in targeted cancer therapy is melanin, a versatile biopolymer that, besides its biological properties (antioxidant, photoprotective, anti-inflammatory and antitumor), possesses intrinsic physicochemical features that make it a reliable nanotheranostic tool with pronounced impact in the oncology field.

**Abstract:**

Cancer poses an ongoing global challenge, despite the substantial progress made in the prevention, diagnosis, and treatment of the disease. The existing therapeutic methods remain limited by undesirable outcomes such as systemic toxicity and lack of specificity or long-term efficacy, although innovative alternatives are being continuously investigated. By offering a means for the targeted delivery of therapeutics, nanotechnology (NT) has emerged as a state-of-the-art solution for augmenting the efficiency of currently available cancer therapies while combating their drawbacks. Melanin, a polymeric pigment of natural origin that is widely spread among many living organisms, became a promising candidate for NT-based cancer treatment owing to its unique physicochemical properties (e.g., high biocompatibility, redox behavior, light absorption, chelating ability) and innate antioxidant, photoprotective, anti-inflammatory, and antitumor effects. The latest research on melanin and melanin-like nanoparticles has extended considerably on many fronts, allowing not only efficient cancer treatments via both traditional and modern methods, but also early disease detection and diagnosis. The current paper provides an updated insight into the applicability of melanin in cancer therapy as antitumor agent, molecular target, and delivery nanoplatform.

## 1. Introduction

Referred to as the “pathology of the century”, cancer remains one of the leading causes of death worldwide [1] despite the tremendous advancements in treatment and prognosis made over the past decades [2,3]. Cancer cells possess the unique ability to escape apoptosis, promote angiogenesis, and activate invasion and metastasis [4,5]. Carcinogenesis results from the accumulation of mutations known as “drivers” that alter cell-division checkpoints [6]. Furthermore, lifestyle-related risk factors such as stress, poorly balanced diet, sedentarism, and tobacco and alcohol consumption have a strong impact on cancer development [2,3]. Apart from the standard oncological treatment methods such as surgery, chemotherapy, and radiotherapy, the development of immunotherapy and gene therapy has significantly improved treatment outcomes [7]. However, assuring treatment specificity, long-lasting efficacy and reduced toxicity remains challenging in all areas of cancer therapy [3,8].

The demand for novel strategies enabling a precise cancer treatment has gained considerable momentum lately [3]. A step forward has been made in this direction by resorting to nanotechnology (NT, the scientific field devoted to the synthesis, characterization, and application of nano-sized materials with dimensions ranging between 1–100 nm [3,9]. NT has revolutionized the way of diagnosing and treating diseases and also manifests great potential for surmounting the current challenges and assisting in the success of cancer therapy [10,11]. The major benefit offered by NT is the targeted delivery of drugs or other therapeutics, enabling disease prevention, diagnosis, and treatment [12]. Additionally, by targeting cancer cells, the tumor microenvironment (TME), or the immune system, several nanoplatforms have been strategically designed for a wide range of cancer therapies to counteract treatment toxicity and lack of specificity, as well as to enhance their efficacy [13]. To date, the oncology field has witnessed the development of a wave of nano-products including liposomes, micelles, dendrimers, and nanoparticles [10].

Recently, melanin burst into the spotlight of modern science as a versatile biopolymer holding extraordinary promise for advanced NT [14]. Deriving from the Greek word “melanos”, meaning “dark”, melanin is the generic term used to evoke the most enigmatic, ubiquitous, and heterogeneous biopigments found in nature [15]. Melanins are widespread among many living organisms (i.e., animals, plants, fungi, and bacteria), and are responsible for the various pigmentations found in human skin, hair, eyes, and brain [15,16,17]. Melanins are divided into eumelanins, pheomelanins, neuromelanins, and allomelanins, which differ from each other in terms of origin, color, structure, and physiological properties [18]. Black eumelanin and yellow-reddish pheomelanin are human and animal pigments derived from the amino acid tyrosine [18,19]. Neuromelanin, a mixture of pheomelanin and eumelanin, is a unique type of mammalian melanin that is not produced by melanocytes, but formed within catecholaminergic neurons via dopamine oxidation [15]. Allomelanins are nitrogen-free pigmented compounds of plant, bacterial, and fungal origin, generally using 1,3,6,8-tetrahydroxynaphthalene as precursor [18,19]. Natural melanin has been defined as a vital biological molecule, serving as a protector against damaging ultraviolet radiation (UVR), reactive oxygen species (ROS), toxins, and metal ions [17,20]. Furthermore, by considering its unique characteristics such as: (i) high biocompatibility, and biodegradability; (ii) broadband light absorption crossing the visible (VIS), ultraviolet (UV), and near-infrared (NIR) spectra; (iii) effective conversion of photon energy into heat; (iv) redox behavior and radical-scavenging properties; (v) paramagnetism and semi-conductivity; and (vi) efficient chelation of organic and inorganic compounds, many melanin-like nanoplatforms have been strategically designed for multiple applications in biomedicine [21,22]. Synthetic melanins include polydopamine (PDA), a nature-inspired polymer and the resulting product of the oxidative polymerization of dopamine. PDA imitates the composition and properties of the natural pigment, being considered a melanin mimetic material [23,24,25,26]. Since its recent discovery, PDA nanostructures have gained popularity as both therapeutic and diagnostic strategies predominantly in the area of cancer management [27].

In the light of existing data, the current paper furnishes an overall glance at the latest research status of melanin and melanin-functionalized nanomaterials in the field of cancer. Starting with a brief description of the biochemical pathways participatory to the synthesis of human melanins, this paper continues by presenting the most exploited therapeutic properties of the pigment. The final part of this article is dedicated to melanin- and PDA-based nanotechnology as a futuristic approach in the targeted treatment of cancer. At the end, future perspectives are discussed.

## 2. Human Melanogenesis in a Nutshell

Melanins, the most enigmatic biopolymers found in nature [18], encompass a widely distributed group of pigments synthetized in human skin, eyes, hair, and nervous system [17]. Melanins are produced by highly specialized cells, the melanocytes, through melanogenesis, a strictly controlled process that is isolated into particular cytoplasmic organelles known as melanosomes in order to avoid the cytotoxicity of some reactive intermediates (i.e., quinones and hydrogen peroxide) [28]. During melanin biosynthesis, melanosomes cover four stages of development, reaching full maturity when filled with melanin [29]. Two types of melanin reside in the epidermal layer of the human skin, namely eumelanin, a black-to-brown insoluble pigment consisting of 5,6-dihydroxyindole (DHI) and 5,6-dihydroxyindole-2-carboxylic acid (DHICA) units, and pheomelanin, a yellow-reddish pigment comprising sulfurous benzothiazine and benzothiazole derivatives [30]. The third type of human melanin, neuromelanin, is a dark, insoluble pigment produced by catecholaminergic neurons of substantia nigra and locus coeruleus, leading to the dark appearance of these brain areas [31]. Neuromelanin results from the oxidation of catecholamines and the subsequent interplay with other cellular components (e.g., metals, proteins, lipids), and accumulates within neuronal cells, since there is no physiological machinery capable of degrading or excreting the pigment [32]. Although the chemical structure of neuromelanin has not been fully defined, it has been discovered that neuromelanin incorporates both benzothiazine and indole units containing a pheomelanin-like core covered by eumelanin at the surface [29,33]. Whether the presence of melanin within neurons is beneficial or toxic remains debatable [29]. On one hand, intraneuronal melanin might act as a shielding factor, protecting the cells from toxins, oxidative stress, and metals. On the other hand, however, its possible involvement in neurodegenerative pathologies (i.e., Parkinson’s disease) has been discussed. Hence, the release of neuromelanin in the extracellular environment during neuronal destruction might trigger pro-inflammatory responses that cause further brain damage [32].

**Figure 1:**
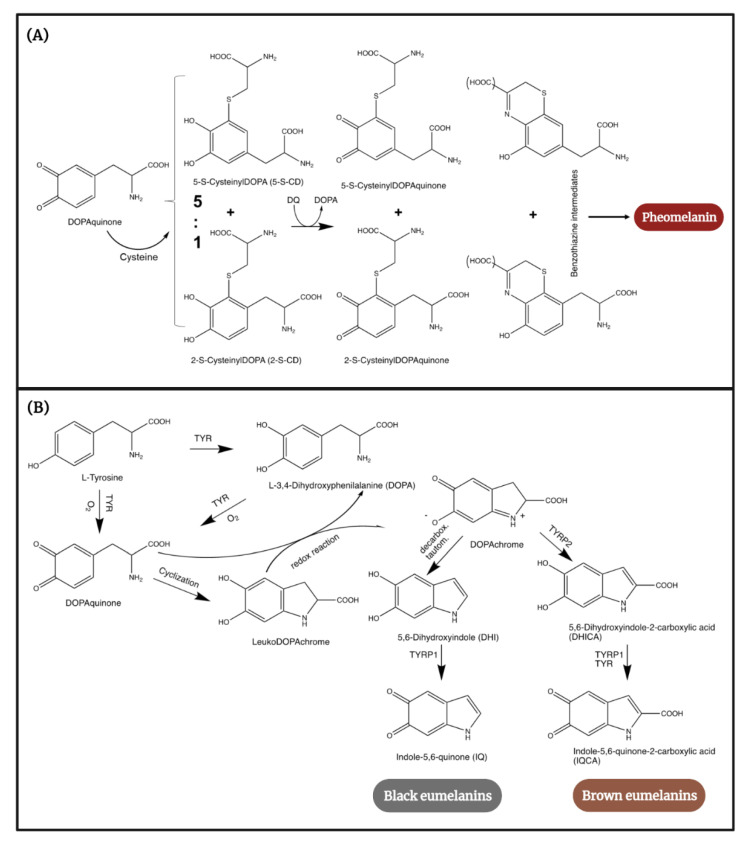
Illustration of the cascade of biochemical reactions participatory to the synthesis of the human melanins (**A**) pheomelanin and (**B**) eumelanin. This image was created using BioRender (BioRender.com). The chemical structures were drawn using ChemDraw 20.1. TYR—tyrosinase, TYRP1—tyrosinase-related protein 1, TYRP2—3,4-dihydroxyphenylalaninechrome tautomerase (Dct).

Cutaneous melanogenesis begins with the hydroxylation of the main melanin precursor, tyrosine, to L-3,4-dihydroxyphenilalanine (DOPA), followed by its oxidation to DOPAquinone (DQ), reactions that are catalyzed by the key enzyme of the process, tyrosinase. Depending on the availability of the substrate, DQ undergoes further modifications directing the process into one of the two possible pathways [34]. When their intracellular levels are elevated, sulfurous compounds such as the amino acid cysteine react with DQ, producing two isomers: 5-S-cysteinyldopa (5-S-CD) and 2-S-cysteinyldopa (2-S-CD). Further oxidation of these isomers leads to benzothiazine intermediates that polymerize to benzothiazole moieties and pheomelanin (Figure 1A) [34,35]. When sulfurous substrates are deficient within melanosomes, DQ spontaneously undergoes an intramolecular cyclization to produce cycloDOPA, which, through a redox process, yields DOPAchrome. Finally, the latter compound rearranges to produce DHI and DHICA units that polymerize to eumelanin (Figure 1B) [35].

**Figure 2:**
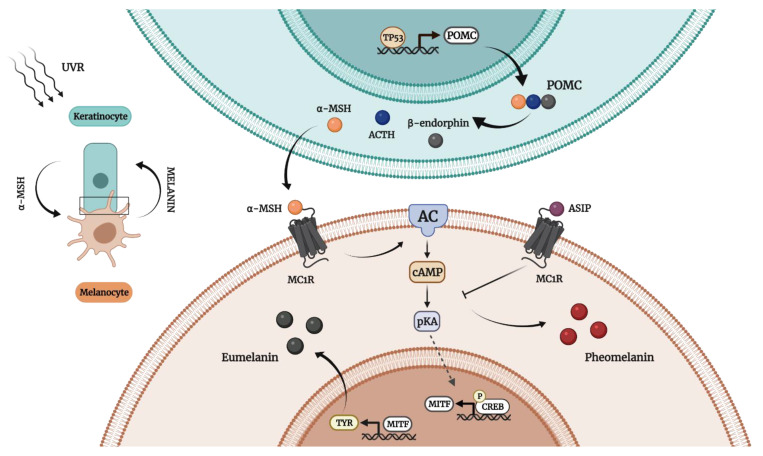
Illustration of the key components in the signaling pathway of melanin synthesis. Keratinocytes trigger epidermal melanogenesis in response to UVR as follows: α-MSH is secreted by keratinocytes and stimulates MC1R, MC1R activates melanogenesis and TYR transcription, TYR promotes eumelanin synthesis, and ASIP blocks MC1R, leading to pheomelanin synthesis. This image was created using BioRender (BioRender.com).

Both melanogenesis and the type of melanin produced depend on a series of factors such as genetic control, hormonal stimulation, enzymes’ translation and proper functioning, reaction media pH, and the substrate available for synthesis. Thereby, the presence of the amino acid cysteine leads to pheomelanin synthesis, whilst its absence encourages the production of eumelanin [34]. Skin pigmentation is controlled by numerous genes involved in the proper functioning of melanocytes, transcription of melanogenic enzymes, and expression of specific receptors [36]. Melanogenesis is regulated via melanocortin-1 receptor (MC1R) signaling [30]. MC1R, a G-protein-coupled receptor expressed on the membrane of melanocytes, is described as a major regulator not only in skin pigmentation, but also in other physiological functions (e.g., control of oxidative stress and genomic integrity) [37]. MC1R’s signaling pathway depends on its interaction with endogenous ligands that are classified as (i) agonists—i.e., α-melanocyte stimulating factor (α-MSH) and ACTH (Adrenocorticotropic hormone (ACTH), or (ii) antagonists—i.e., Agouti signaling proteins (ASIP) [30]. Binding of an agonist to MC1R triggers melanogenesis in a cyclic adenosine monophosphate (cAMP)-dependent manner [36]. MC1R stimulation leads to adenylate cyclase (AC) activation, increasing the intracellular levels of cAMP [34]. Through protein kinase A (pKA), cAMP promotes the phosphorylation of the cAMP response element binding (CREB) protein, which stimulates microphthalmia-associated transcription factor (MITF) overexpression. The final step of this cascade of reactions is the transcription of tyrosinase [34]. Tyrosinases are highly heterogeneous and widespread enzymes that catalyze, using copper as a cofactor, the hydroxylation of monophenols and subsequent oxidation of diphenols to quinones [30]. The MC1R activation also increases the melanosomal pH from acidic to neutral values, at which the catalytic efficiency of tyrosinase and eumelanin synthesis are enhanced [35]. On the other hand, the ASIP–MC1R interaction reduces the cellular cAMP levels, inhibiting MITF expression and tyrosinase translation, which finally leads to pheomelanin synthesis [30]. In fact, ASIP competitively blocks the binding locus for α-MSH [30], acting as an inverse agonist [35]. Once formed, melanin can be either retained within melanocytes or transferred to other cells. For instance, skin melanosomes containing melanin are transported to the surrounding keratinocytes, whereas in the retinal pigment epithelium of the eyes, melanin is preserved within melanocytes [29]. Upon UV irradiation, keratinocytes trigger melanogenesis by secreting promelanogenic compounds including α-MSH, ACTH [30], and β-endorphin [36], which result from the proteolysis of a multicomponent precursor polypeptide encoded by the pro-opiomelanocortin (POMC) gene (Figure 2) [38].

Additionally, keratinocytes produce prostaglandin E2 (PGE2), endothelin-1 (ET-1), fibroblast growth factor (FGF), granulocyte macrophage colony stimulating factor (GM-CSF), thus controlling the growth, proliferation, and activity of the melanin-producing cells [30]. ET-1 acts as a “survival factor”, enhancing the capacity of melanocytes to withstand stress during and after UV exposure [36], supports the proliferation and migration of melanocytes, and stimulates melanin production and DNA repair, reducing apoptosis in the UV-affected cells [30,36]. E2 prostaglandins are abundantly secreted by keratinocytes upon UV irradiation, playing a key role in dendritogenesis and cAMP signaling control [30]. Moreover, keratinocytes are able to synthesize the active form of vitamin D, which, along with the other paracrine factors discussed above, enhances the ability of melanocytes to respond to α-MSH stimulation and thus increase the production of eumelanin [36].

## 3. Therapeutic Properties

Melanin manifests a host of pharmacological properties and has received considerable attention lately as a potential therapeutic agent [17]. Compared to conventional drugs, melanins possess numerous advantages, including a broad spectrum of action and low toxicity. The biological functions of melanin are linked to its physicochemical properties [39]. First, melanin is insoluble in water, affecting its bioavailability following administration [40]. Actually, melanin has a low solubility in most organic and inorganic solvents, except for aqueous alkaline solutions [33,41]. The absorption spectrum of melanin shows an exponential wavelength dependence and extends from the UV to the VIS and NIR regions, owing to the complex and heterogeneous structure of the biomolecule [42]. The semi-conductivity of melanin also provides a plausible explanation for its broad optical absorbance [43]. Alkaline melanin solutions possess strong absorbance in the UV domain with a maximum absorption at wavelengths ranging from 196 to 300 nm, depending on the source, that progressively decreases at longer wavelengths. In regard to the VIS spectrum, epidermal melanin displays different mechanisms of absorption, mainly due to its existence in both particulate and soluble forms [33]. The absorbance of particulate melanin shows a linear increase from 800 to 400 nm, while the absorbance of soluble melanin increases exponentially from 600 to 300 nm and resembles the spectrum of the skin pigment [44]. Melanin was also described as possessing a paramagnetic character that might affect its interactions with drugs and metal ions [45].

The most important therapeutic properties of melanins obtained from various sources are presented in Table 1.

### 3.1. Antioxidant Effect

Both natural and synthetic melanins exhibit a redox behavior [17,35], counteracting ROS such as superoxide, hydroxyl radical, and singlet oxygen [57]. However, a conflict of interest occurs between eumelanin and pheomelanin, since one behaves as a free radical scavenger while the other has pro-oxidant effects [17]. The antioxidant activity of eumelanin is strictly related to its chemical structure (i.e., phenolic and indolic groups [58]), as well as to the DHI/DHICA ratio [57]. Therefore, the eumelanin pigments structured mostly from DHICA units show a stronger antioxidant property in contrast to those formed of DHI chains that, in fact, exhibit cytotoxic effects toward melanocytes through ROS generation. The pro-oxidant effects of DHI-eumelanin are completely countered by the DHICA moiety [57]. The explanation for these differences is attributed mostly to the extra carboxylic radical found in the acidic DHICA fraction. The negative charge of the carboxylate groups linked to the pyrrole ring of the DHICA units generates a non-planar and partly linear configuration that is unable to form π-stacked supramolecular aggregates. Formation of weak aggregates leads to a greater accessibility for free radicals. The redox properties of eumelanin are also attributed to the transition of the indole units from the catechol to the quinone state and vice-versa, as well as to the hydrogen atom transfer (HAT), which offers a potent hydroxyl radical-scavenging ability [35]. Moreover, the carboxyl units offer some peculiar features to the DHICA units, such as a reduced number of reactive sites available for polymerization and cross-linking and a lower oxidation potential, making the macromolecule less susceptible to further modifications [59]. The molecule’s polymerization reduces the number of hydroxyl groups involved in HAT scavenging, and thus, its antioxidant activity [57]. These facts are supported by Jiang S and co-workers, who showed that only DHICA-eumelanin exhibits a potent •OH scavenging activity in the Fenton reaction, whereas DHI-eumelanin alone acts as a pro-oxidant compound. Moreover, the mixture of the two types of eumelanin diminished the oxidant effect of the DHI fraction, suggesting that the intracellular DHI–DHICA association is necessary to reduce the oxidative damage within melanocytes [57].

The antioxidant activity of the melanic pigment results from the association of the two opposite effects and due to the fact that eumelanin represents the predominant fraction within the human skin [17]. A series of experiments were conducted in order to highlight the antioxidant character of melanins from different sources. El-Naggar N and El-Ewasy SM tested the ability of a *Streptomyces sp.* purified melanin to neutralize the 2,2′-azino-bis (3-ethylbenzothiazoline-6-sulfonic acid) (ABTS) radical. Their results revealed that the antioxidant capacity of melanin is comparable to that of ascorbic acid used as standard. According to the authors, melanin exhibited a potent scavenger activity against ABTS, at the concentration of 100 μg/mL, through hydrogen transfer [48]. Kumar et al. compared the free radical scavenger potential of natural melanin extracted from *Aspergillus bridgeri* to that of synthetic melanin and ascorbic acid using the 2,2-diphenyl-1-picrylhydrazyl (DPPH) assay. Their work shows that the melanins from both sources exhibit a significant and concentration-dependent antioxidant activity, but are less efficient than the standard [60]. Yao and co-workers conducted a complex study to reveal the antioxidant character of some melanin fractions extracted from chestnut shell against specific ROS such as the hydroxyl radical and the superoxide anion. They also tested their potential to inhibit the lipid peroxidation and to scavenge the DPPH radical. While melanin exhibited a strong capacity to inhibit lipid peroxidation and manifested a dose-dependent quenching activity towards •OH and DPPH, it acted as a weak antioxidant against superoxide anions even at high concentrations [58].

### 3.2. Photoprotective Capacity

Exposure to UVR triggers a variety of harsh cutaneous responses, including inflammation, immunosuppression, oxidative stress, and DNA damage. UVR is subdivided into three main components: UVA (315–400 nm), UVB (290–320 nm), and UVC (<290 nm) [61,62]. While UVC light is filtered out by atmospheric ozone, UVA (~95%) and UVB (~5%) radiation reaches the earth’s surface and initiates toxic events in human skin such as photoaging and carcinogenesis [61,63]. Skin penetration of UVR is wavelength-dependent [61]; hence, UVA photons penetrate deeply into the skin layers, reaching the dermis, while UVB possesses a superficial skin penetration ability, being almost completely absorbed by the epidermal layer [61,64]. UVA-induced phototoxicity is related to its ability to generate a massive production of ROS, while UVB photons cause direct DNA damage and molecular rearrangements, forming cyclobutane pyrimidine dimers (CPDs) and 6–4 photoproducts (6–4PPs) [61,65].

Melanin stands as the first-line defense strategy against UVR-induced skin injury by hindering the penetration of UV light and converting it into harmless heat [64]. Furthermore, UVR stimulates skin tanning, which provides additional photoprotection by reducing the formation of DNA photoproducts following UV irradiation [66,67]. Melanin acts as a physical barrier, scattering up to 75% of the UV radiation [68], and possesses a sun photoprotection factor (SPF) value ranked between 1.5 and 2.0 [69]. However, the photoprotective role is highly dependent on the melanin type. Eumelanin exerts a higher efficiency in absorbing UV photons than pheomelanin [61]. Eumelanins of both natural and artificial origins show a broadband absorption within the UV spectrum [70] and possess antioxidant activity [20]. Among the eumelanin units, DHICA-eumelanin confers stronger photoprotection than DHI-eumelanin [67]. The underlying mechanisms of the UV protective effect exerted by eumelanin still remain an unsolved enigma. However, Corani et al. provided some interesting discoveries in this regard. According to their study, the unique ability of eumelanin to dissipate harmful UVR as heat is a specific property of coupled DHICA components and is dependent on the interunit bonding patterns within the oligomer and polymer chains [70].

In contrast to its counterpart, pheomelanin has been classified as a photosensitizing chromophore, possessing weak shielding capacity against UVR, ROS-generating ability when irradiated, and a potential carcinogenic property [67,71].

### 3.3. Anti-Inflammatory Properties

A strong correlation has been already established between acute or chronic inflammation and skin pigmentation [72]. In particular, inflammatory mediators (i.e., interleukins IL-1, -4, -6, -18, and -33) trigger melanin overproduction or abnormal cutaneous deposition, leading to post-inflammatory hypermelanosis by either promoting melanogenesis or by regulating the melanocytes [72,73]. Conversely, recent studies report the potential of melanic pigments to influence the levels of inflammatory markers and reduce inflammation. For instance, a research group investigated the melanin produced by the *Nadsoniella nigra* strain X-1 in terms of in vivo anti-inflammatory effect in hepatic disease. Their studies show that melanin is able to lower the serum content of IL-1 and tumor necrosis factor alpha (TNF-α) expression, restore the levels of IL-10 and transforming growth factor-β (TGF-β) cytokines to control values, and prevent nuclear factor-κB (NF-kB) activation in hepatocytes [74,75]. In another publication, by Kurian et al., melanin isolated from marine *Bacillus spp.* BTCZ31 inhibited, in a dose-dependent manner, the activity of three enzymes—cyclooxygenase (COX), lipoxygenase (LOX), and myeloperoxidase (MPO)— involved in inflammatory responses in RAW 264.7 murine macrophage-like cells. In addition, melanin decreased the intracellular nitrite levels and showed a 67.55% scavenging activity in the DPPH assay at 100 μg/mL [76].

### 3.4. Anticancer Activity

It has been traditionally known that skin pigmentation is the predominant factor protecting melanocytes and other epidermal cells from UV-induced carcinogenesis [68,77]. Additionally, recent studies suggest the possible implication of melanin in the behavior of malignant melanoma, the most aggressive and lethal skin cancer type [78,79]. Melanin pigmentation is highly deregulated within melanoma cells, which can easily switch between pigmented and non-pigmented states. Intriguingly, melanoma cells are not able to excrete the pigment like normal melanocytes, becoming heavily pigmented. The main solution for lowering the intracellular amount of melanin refers to consecutive cell divisions [80]. Moreover, it has been revealed that during melanogenesis, the melanoma cells become less aggressive because a melanoma cell either migrates or synthesizes melanin [80,81]. Further, amelanotic (or non-pigmented) melanoma is associated with a poorer patient survival rate and spreads more than the pigmented melanoma [80]. Sarna’s research group manifested a great interest in the correlation between the melanin amount within the cells and their invasive character. In that matter, they first conducted in vitro experiments on amelanotic and melanotic Bomirski hamster melanoma cells, studying the melanin content within the cells, their proliferative ability, morphology, and cytoskeleton organization, as well as their nanomechanical properties. Their study unveiled that the intracellular presence of melanin reduced the cells’ deformation capabilities and thus their ability to penetrate through a membrane or an endothelial barrier. Even though the cell cytoskeleton is the main contributor to the cellular mechanics of normal and cancer cells, according to the authors, in the case of melanoma cells, the presence of melanin instills unique mechanical properties that affect the behavior of melanotic cells [81]. Their further analysis of the role pigmentation plays in the abilities of melanoma cells to spread throughout the body was performed in vivo in nude melanoma-inoculated mice. The results regarding the characteristics of the melanoma SK-MEL-188 cells (i.e., melanin content, nanomechanical properties) prior to inoculation led to conclusions similar to those of their previous paper [81]. Briefly, the in vivo experiments revealed that the livers from mice inoculated with non-pigmented melanoma cells developed the highest number of metastatic tumors when compared to mice inoculated with pigmented melanoma cells and were significantly heavier than the latter. These results strengthened their belief that melanin represents a determining factor in the metastatic behavior of melanoma [80].

**Figure 3:**
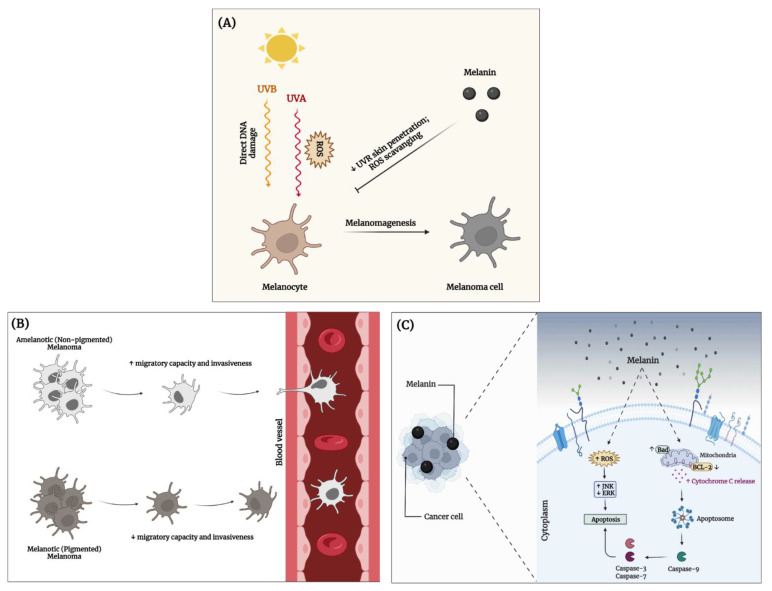
Melanin implications in cancer development. (**A**) Cancer prevention—melanin counteracts UVR-induced damage and melanomagenesis; (**B**) cancer metastasis—differential metastatic potential of melanoma cells depending on the presence of intracellular melanin; and (**C**) cancer treatment—melanin exerts a pro-apoptotic effect against cancer cells. This image was created using BioRender (BioRender.com). ↑ increased; ↓ decreased.

The anticancer properties of melanins from various sources was examined in both in vitro and in vivo studies. For instance, melanoid pigments extracted from a *Streptomyces glaucescens* strain exhibited a strong antiproliferative activity against a skin cancer cell line (HFB4) and showed a less cytotoxic effect against non-cancerous cells, human lung fibroblast (WI-38) and human amnion (WISH), even at high concentrations [48]. Another in vitro study revealed that *Nigella sativa*-derived melanin inhibited the proliferation of two colorectal adenocarcinoma cell lines (HT-29 and SW620), and exerted a pro-apoptotic effect by activating the intrinsic mitochondria-dependent apoptotic pathway, c-Jun N-terminal kinase (JNK) pathway, and caspase-3/-7, and by inhibiting the expression of B-cell lymphoma 2 (Bcl-2) family proteins, as well as the extracellular signal-regulated kinase (ERK) activity partially via toll-like receptor 4 (TLR4) [53]. Shi F and contributors investigated the in vivo antitumor effect of melanin extracted from the *Lachnum* fungus and its arginine derivatives on hepatocarcinoma-bearing mice, revealing their ability to significantly inhibit the tumor growth. Moreover, they showed no distinct systemic toxicity to major organs (liver and kidney) in the tumor-bearing mice. Further experiments demonstrated that the natural melanin and the arginine-derived melanins regulated the hepatic and renal functions, enhanced the serum concentration of IL-2, IL-6, TNF-α, and interferon gamma (IFN-γ), while decreasing the VEGF (vascular endothelial growth factor) and basic fibroblast growth factor (bFGF) levels. Thus, the authors propose that the mechanism of the anticancer effect of melanin is based on its capacity to improve the immune functions, induce apoptosis, and inhibit angiogenesis [82]. Based on the currently known data, the main role of melanin in cancer prevention, metastasis, and treatment are illustrated in Figure 3.

## 4. Melanin-Based Targeted Cancer Therapy

### 4.1. Melanin Targeting in Cancer Therapy

Targeted cancer therapy (TCT) is an advanced treatment method utilizing therapeutic agents designed to interfere with specific molecular targets expressed within cancer cells (e.g., signaling molecules, growth factors, apoptosis modulators, and cell-cycle proteins) in order to restrain tumor growth and progression [83,84]. Melanin became an extensively exploited biomolecule as potential target in TCT, enabling a selective tumor treatment with therapeutics possessing affinity for the pigment [85]. Since melanin presence was detected in the majority of malignant melanoma (MM) primary tumors (90%), with the predominant fraction being represented by eumelanin [86,87], melanin-targeted therapy emerged as an interesting approach in combating MM. However, considering its presence within healthy pigmented cells (i.e., melanocytes), its selectivity and biocompatibility should be evaluated. One of the first studies in this direction showed that the polycyclic aromatic compound methylene blue accumulates preferentially within MM cells owing to its melanin-binding properties and serves as a carrier for radioisotopes (i.e., ^131^I, ^211^At), thus allowing targeted radiotherapy [88]. In a more recent study, Degoul et al. demonstrated potent antimelanoma efficacy with an ^131^I-labeled heteroarylcarboxamide molecule ([^131^I]ICF01012) associated with a lack of toxicity towards pigmented organs [89]. Similarly, the results of a study conducted by Viallard et al. indicated the ability of [^131^I]ICF01012 to inhibit tumor growth and to increase the survival rate of melanoma xenograft-bearing mice following treatment. Furthermore, they showed that the uptake of ICF01012 labeled with iodine-123 (^123^I) is dependent on the melanin content [90].

Based on its significant reactivity with amino groups, in a recent study, PDA was deposited on the membrane of cancer cells, serving as “artificial receptor” for the targeted delivery of two anticancer drugs (i.e., cisplatin and saporin). PDA-specific generation on tumor cells was facilitated by the characteristics of the tumor microenvironment (high K^+^ and H_2_O_2_ levels). The results revealed that PDA production exerted no toxicity against CCRF-CEM T lymphoblastoid cells, indicating excellent biocompatibility. However, its presence elevated the anticancer efficiency of both drugs. Furthermore, by comparing cancerous MDA-MB-231 with non-tumoral MCF10A breast cells, the authors observed an obviously higher cytotoxicity of cisplatin against tumor cells following PDA deposition [91].

**Figure 4:**
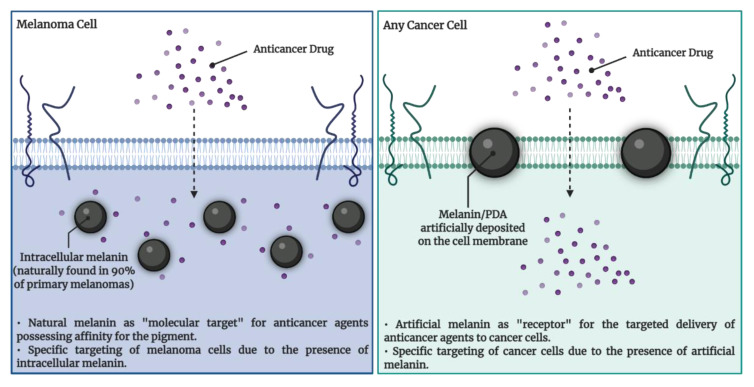
Natural and artificial melanins as targets in cancer therapy. This image was created using BioRender.com.

Guiding antitumor agents towards cancer cells based on (i) their affinity for melanin and (ii) the chelating properties of the pigment represents an encouraging approach for TCT. In the case of malignant melanoma, which is generally pigmented, anticancer compounds accumulate within the cells by targeting the already existing intracellular melanin. Regarding other cancer types, the artificial deposition of melanin on cancer cells is essential to facilitate a pigment-targeted treatment. A schematic illustration is presented in Figure 4.

### 4.2. Melanin Nanoparticles (MEL-NPs) in Cancer Therapy

#### 4.2.1. Synthesis of MEL-NPs

MEL-NPs can be produced both by biological extraction, which resorts to the direct separation and purification of spherical NPs from natural sources, and chemical synthesis. For instance, Le Na et al. obtained nanomelanin particles by dissolving commercial melanin powder in alkaline solvents (i.e., sodium and/or ammonium hydroxide) and neutralizing the solution using hydrochloride acid [92]. Artificially synthesized polydopamine nanoparticles (PDA-NPs) mimic natural melanin, possessing similar properties but provide better controllability. Another advantage of PDA-NPs is their simple preparation protocol. PDA-NPs are routinely produced through the oxidative polymerization of dopamine monomers under alkaline conditions, resulting in sphere-like shaped particles in sizes ranging from 100 to 500 nm [93,94,95]. Conclusive TEM and SEM images of spherical PDA-NPs of different sizes were presented in a review by Yue et al. [93]. Various methods can be applied for PDA synthesis, such as solution oxidation (the most common), enzymatic oxidation, and electropolymerization [96]. Generally, PDA is synthesized in a water–ethanol environment and by using ammonia as a catalyst. It has been found, however, that some proteins (e.g., human serum albumin), and surfactants (e.g., sodium dodecyl sulfate, hexadecyltrimethylammonium bromide) accelerate dopamine oxidation and PDA production rates [25]. Furthermore, in a recent paper, Lemaster et al. reported an advantageous and rapid synthesis method for ultrasmall melanin nanoparticles (<50 nm) at acidic and neutral pH values (6.4–7.0) via dopamine polymerization under UV irradiation, which accelerates the oxidation process due to ROS generation. Interestingly, dopamine polymerization is highly dependent on the pH value, UVR presence, and reaction time. Therefore, the authors noted the following aspects: (i) polymerization increased as UV irradiation time increased, (ii) at pH values ranging from 8.0 to 10.0, dopamine polymerizes to form synthetic MEL-NPs with or without UVR, and (iii) UVR is necessary for dopamine polymerization under pH conditions of 6.4 [94].

#### 4.2.2. Applications of MEL-NPs in Cancer Therapy

**Figure 5:**
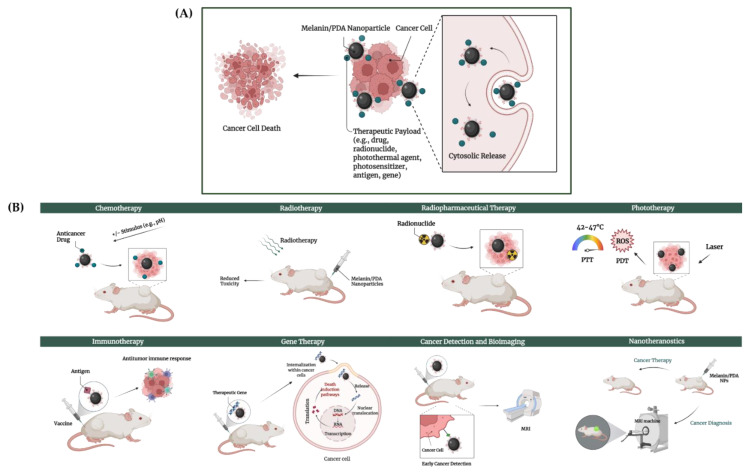
(**A**) Targeted delivery of therapeutics to cancer cells by melanin/PDA nanoparticles, and (**B**) main applications of melanin/PDA nanoparticles in cancer therapy. This image was created using BioRender.com.

In the last decade, both natural and artificial MEL-NPs, including PDA-NPs, have been widely employed in the design of novel platforms for biomedical applications [97]. Focusing on cancer therapy, the promising results of the melanin-based nanotechnology reported in recent studies are further presented in Table 2 and Figure 5.

##### Chemotherapy

Chemotherapy represents the pharmacological approach in cancer treatment referring to the administration of cytotoxic drugs [133]. The therapeutic efficiency of drugs depends heavily on their ability to reach the intended site of action [134]. The hallmark of the majority of bioactive agents is related to their inconvenient properties (e.g., poor water solubility and bioavailability, nonspecific distribution throughout the body, transient blood circulation time, severe systemic adverse events, and occurrence of multidrug resistance following long-term treatments), leading to an unfavorable outcome in therapy. The emergence of nanotechnology provided a state-of-the-art perspective regarding drug-based therapy with the development of custom-designed delivery systems [19]. Drug delivery platforms are engineered nano-carriers able to load, transport, and release bioactive compounds at targeted tissues in a controlled fashion. Delivery devices provide multiple therapeutic benefits such as: (i) drug protection against physicochemical or enzymatic degradation, (ii) enhanced bioavailability and therapeutic effect, and (iii) reduced side effects and dosing frequency during treatment [134]. The chemical synthesis of melanin provides control over the size, surface characteristics, loading, and drug-release efficiency of the obtained nanoplatforms. Melanin is able to bind drugs possessing aromatic structure through π–π stacking [135]. Alternatively, pharmacologically active molecules can be linked to the surface of melanin through covalent bonds due to its abundance in functional groups (i.e., o-quinone, amine, catechol, imine), or be simply encapsulated within the polymer matrix via non-covalent bonding [136].

Melanin nanocarriers have been explored mostly in the area of cancer therapy due to their distinguished drug-binding properties, as well as innate antitumor activity. Various in vitro and in vivo studies indicate the anticancer effects exerted by melanins (Section 3.4). PDA also behaves as an antineoplastic system, selectively killing tumor cells without causing toxicity to healthy cells [97,137]. Perring et al. showed that water-soluble melanin NPs induced selective cytotoxicity in human rhabdomyosarcoma (RH30, and RD) and glioblastoma (U-87 MG and Mo59K) tumor cell lines via iron deprivation, which is due to the chelating properties of melanin polymers [138]. In another study, Gabriele et al. reported that highly monodispersed uncoated and glucose-coated MEL-NPs are massively absorbed by malignant cancer cells and influence their viability, which reduces with increasing numbers of absorbed NPs [139]. With the aim of achieving a targeted delivery of paclitaxel (PTX) in osteosarcoma therapy, Zhao et al. coated PTX-NPs with PDA and functionalized them using alendronate (ALN) as ligand. The obtained system showed stability in physiological media, lacked hemolytic potential, and exhibited greater cytotoxicity on K7M2 wt osteosarcoma cells compared to PTX-NPs [99]. In order to diminish the effective dose and side effects of a current oncologic drug used in the treatment of HER2+ breast cancer, Nieto et al. incorporated PTX into PDA-NPs that were subsequently decorated with trastuzumab and validated the antitumor efficiency of the novel nanoplatform in 2D and 3D in vitro models. The results revealed that the NPs reduced the viability rate and increased the number of early apoptotic HER2+ breast tumor cells in a trend similar to that obtained with equivalent concentrations of free PTX, suggesting that the loading into PDA-NPs does not affect the pharmacological activity of the tested drug. Similar conclusions were drawn following the testing on 3D cultured cells, where it was shown that PDA-NPs disintegrated and reduced the viability of HER2+ breast tumor spheroids. Furthermore, the authors showed that the charged PDA-NPs reduced the viability of stromal cells to a lesser extent compared to similar concentrations of PTX [100]. 

A breakthrough in nanotechnology-based cancer treatment has been the development of stimuli-responsive NPs, which exceed conventional delivery systems due to their ability to release drugs in response to external (e.g., radiation, electromagnetic, thermal) or internal (e.g., pH, enzyme, ROS, hypoxia, redox) stimuli [140]. Recent reports describe the utilization of PDA for the purpose of stimuli-responsive drug delivery. For instance, Wu et al. developed RGD-modified polydopamine-paclitaxel-loaded poly (3-hydroxybutyrate-co-3-hydroxyvalerate) NPs, in which PDA served as a pH-sensitive coating able to enhance drug stability and avoid premature drug release. This nanosystem showed a higher PTX release rate in vivo at pH values of 5.0–6.5 (which are specific for inclusion bodies and lysosomes of cancer cells) than at physiological pH (7.4), suggesting an intelligent, controlled, and pH-dependent drug release [101]. An explanation in this regard might come from the ability of PDA to dissolve slowly in acidic conditions [141]. Furthermore, the NPs improved the PTX solubility and enhanced its antitumor effect, while showing favorable biocompatibility [101].

##### Radio(pharmaceutical) Therapy

Radiotherapy, which relies on high-energy ionizing radiation to kill tumor cells, is a conventional cancer treatment strategy widely employed in clinical practice [142]. Radiation exposure generates free radicals capable of destroying not only the tumor, but also the normal tissue despite the ability of cells to produce self-protective molecules (i.e., glutathione and metallothionine). Research later oriented towards the development of efficient radioprotectors, and nanotechnology seems a promising strategy to overcome radiation-induced side effects in patients [143]. Melanins show broadband radiation absorption in the UV-VIS-NIR spectra, while MEL-NPs possess similar properties [22]. In this regard, Huang et al. demonstrated that synthetic MEL-NPs confer photoprotection to epidermal keratinocytes (HEKa) against UV-induced damage in a fashion similar to natural melanosomes [144]. Schweitzer et al. showed that the systemic administration of melanin-covered NPs reduced the hematologic toxicity in CD-1 mice exposed to external radiation or radioimmunotherapy [107]. In addition, another study revealed that the pre-treatment of Chinese hamster ovary cells with MEL-NPs (6.25, 12.5, 25 and 50 µg/mL) attenuated gamma radiation-induced cytotoxicity [108].

Radiopharmaceutical therapy (RPT) has been described as a novel, safe, and effective treatment modality with wide applicability in the management of various neoplasms (e.g., thyroid, breast, prostate, lung, etc.) [145,146]. Owing to its multidisciplinarity, RPT sits at the junction of pharmacology, oncology, radiochemistry, radiobiology, cell biology, diagnostic imaging, and physics [145,147]. In contrast to radiotherapy, where the administered radiation is external, in RPT the radiation is systemically or locally transported by pharmaceuticals that preferentially accumulate within cancer cells, enabling a tumor-targeted toxicity while preserving the nearby healthy tissues [145,146]. The most preferred ionizing radiation types are α-particles and electrons [146]. As a third type, photons are not suitable for the localized delivery of cytotoxic radiation, but are useful in RPT imaging [145].

Zhong et al. showed that the conjugation of both radionuclides (^131^I) and an anticancer drug (doxorubicin) to PEGylated PDA-NPs enables the efficient radio-chemotherapeutic treatment of breast cancer in vivo. The combined treatment modalities offered superior results compared to the respective monotherapies [110]. In a similar fashion, Li et al. obtained a synergistic repression of 4T1 tumor growth in mice following the administration of PDA-PEG NPs carrying ^131^I, sanguinarine, and metformin [109].

##### Phototherapy

Over the last few years, photothermal therapy (PTT) and photodynamic therapy (PDT) have gained enormous attention as minimally invasive alternative strategies in cancer treatment, given their efficiency in overcoming chemotherapy-related toxicity as well as tumor resistance to medication. Such phototherapies rely on the administration of photothermal agents (PTAs) (e.g., polypyrrole, polyaniline) or photosensitizers (PSs) (e.g., porphyrins, phthalein cyanogen, polyacrylamide, silica) that generate heat or ROS upon illumination [148,149,150].

PTT is a treatment method based on selective tumor ablation through hyperthermia (temperatures between 42 and 47 °C), subjecting cancer cells to thermal stress, which initiates apoptosis [111,150,151]. PTT exploits photothermal PTAs capable of inducing heat within the tumor environment when exposed to NIR light, without causing harm to the surrounding normal tissue. It could be assumed that PTT is somehow a branch of nanomedicine, since it involves the use of nano-scaled PTAs that vary from inorganic to polymeric nanoparticles [97,152]. The key element of the photothermal property is the conversion of electronic excitation energy by light-excited molecules into vibrational energy and heat [153]. Regardless of the exciting progress made in this realm, few of the currently available PTAs have reached clinical application due to their poor water solubility, dissatisfactory surface functionalization, reduced photothermal conversion efficiency, and long-term safety concerns [19]. Melanin NPs fulfill the main requirements of an ideal PTA, such as a large absorption coefficient in the NIR spectrum and enhanced photothermal conversion power that exceeds that of other reported PTAs [93,97]. For instance, Liu and collaborators showed that dopamine–melanin colloidal nanospheres (Dpa–melanin CNSs) displayed a much higher photothermal conversion efficiency (40%) compared to gold nanorods (22%) which are widely evaluated for cancer therapy [154]. Another advantage retained by MEL-NPs is their easy surface modification, which additionally might enhance their PTT potential. As a selected example, Yang et al. showed that tailoring of PDA-based synthetic MEL-NPs with arginine increases their NIR light absorption and PTT performance both in vitro and in vivo [111].

Another phototherapeutic technique that has been extensively explored in cancer treatment is PDT. The PDT mechanism of action involves the interaction between light, a PS, and molecular oxygen, resulting in photo-physical-chemical reactions and production of ROS that oxidize cellular components, making cancer cells unviable [151]. The PDT procedure requires several steps, as follows: (i) topical or systemic administration of the PS and its accumulation within the target tissue; (ii) exposure of the target tissue to light at an appropriate wavelength; and (iii) photodynamic reaction between the excited PS and surrounding oxygen, leading to ROS generation. A proper PS should be a single, highly purified, and stable chemical with a well-defined molecular structure, that possesses a superior photosensitizing ability, exhibits no long-term toxicity, accumulates preferentially within the concerned tissue, and undergoes fast body clearance to prevent prolonged photosensitivity [149]. The versatile surface chemistry of PDA has motivated the conjugation of photosensitizers on PDA-NPs [155]. Yan et al. generated a PDA-based nanomedicine modified by folate and loaded with a cationic phthalocyanine-type photosensitizer (PDA-FA-Pc) for the goal of eradicating cervical and breast tumors through PDT. The resulting nanocarrier showed high stability in physiological media, release of the photosensitizer in tumor-specific acidic conditions, increased uptake and phototoxicity in tumor cells (Hela and MCF7) compared to healthy cells (HELF and L02), and inhibited tumor growth with no obvious systemic toxicity in xenograft-bearing mice [119].

Recent reports demonstrated that the synergistic effect resulted from the association of PTT with PDT in tumor therapy. An explanation in this regard comes from the ability of PTT to increase the intratumor blood flow through vasodilatation, leading to an enriched oxygen supply, which is essential for PDT and ROS generation [148]. Zhang et al. designed chlorin e6 conjugated PDA (PDA-Ce6) nanospheres as dual-modal therapeutic agents for simultaneous PTT and PDT against hepatocellular carcinoma. PDA-Ce6 nanospheres showed enhanced internalization within HepG2 cells, higher PDT and PTT efficiency, as well as increased ROS generation compared to free Ce6. In tumor-bearing nude mice, the combined PDT/PTT treatment exerted a stronger anticancer effect over any single modality treatment, resulting in tumor regression [120].

Thereafter, straightforward approaches illustrated the significance of MEL-NPs as both PTT and PDT agents, providing a personalized treatment option with increased therapeutic efficacy.

##### Immunotherapy

Immunotherapy (IT) has transformed the field of oncology by changing the concept of cancer treatment from drugging cancer cells to stimulating the body’s own immune system to fight against cancer cells [156,157]. The immune system plays a chemoprotective role by identifying neoplasia in incipient stages, as well as eliminating tumors. However, tumors are endowed with the ability not only to avoid immune surveillance, but also to exploit the immune system to further grow and metastasize [158]. Depending on the effect induced towards the host’s immune system (suppression or activation), two types of IT were identified, namely immunosuppressive and immunostimulatory therapies. Immunostimulatory therapy acts by inducing a strong antitumor immune response, leading to tumor eradication, as well as disease recurrence prevention, and is currently applied in cancer treatment [156]. Cancer IT faces several challenges such as increased immune-mediated toxicity, as well as an ineffective and non-targeted delivery of immunostimulating agents to immune cells. Nanotechnology emerged as a solution to overcome these limitations, opening up a novel approach in combating cancer [156]. NPs enhance cancer IT either by improving the efficiency of cancer vaccines, or by modulating the tumor microenvironment (TME) to facilitate immune activation [159].

Owing to its abundance of catechol groups, PDA has been shown to covalently link free amine and thiol groups in antigens for their effective delivery in cancer IT. Wang et al. used PDA as a carrier for the tumor lysate derived from MC38 cells as a potential nanovaccine (TCL@PDA NPs) for colorectal cancer IT. The authors showed that TCL@PDA NPs delayed cancer progression in tumor-bearing mice, promoted antigen uptake, bone-marrow-derived dendritic cell (BMDC) maturation, and Th1-related cytokine secretion, while conferring long-term protection against tumors via CD4+ and CD8+ T cell enhancement. Interestingly, the antitumor effect was ascribed as well to empty PDA-NPs whose ability to modulate the maturation of dendritic cells (DCs), facilitate the production of activated T cells, and decrease the subpopulation of myeloid-derived suppressor cells (MDSCs) within the TME was demonstrated [121]. Similarly, in another study, PDA-NPs were designed as carriers of the tumor model antigen ovalbumin (OVA) in colon cancer therapy. The prepared vaccine demonstrated a lack of cytotoxicity against BMDCs, elevated cellular uptake, easy migration to lymph nodes, as well as enhanced CD8+ T cell-mediated immune response, which led to the suppression of tumor growth [123].

NPs may also act as mediators of tumor ablation via PTT or PDT, or as radiosensitizers, increasing the release of immunostimulating agents and thus boosting the body’s immune system [156]. In a very recent paper, Huang et al. integrated mesoporous silica NPs (vector), PDA (photothermal agent), and ammonium bicarbonate (antigen release promoter) in a single nanoplatform for the efficient delivery of OVA in melanoma PTT-IT combined treatment. PDA coating conferred excellent light-to-heat converting properties following NIR exposure. The nanovaccine administration increased DC activation and maturation, enhanced CD8+ and Th1 CD4+ T cell responses, and eradicated primary tumors, while preventing their recurrence in mice [122].

##### Gene Therapy

With the enthusiastic advancements made in molecular biology, the concept of gene therapy (GT) gradually became a promising strategy for the specific treatment of human diseases caused by genetic anomalies, cancer included. GT refers to the transfer of genetic material (e.g., DNA, RNA, gene sequences) into pathological cells with the purpose of intentionally altering gene expression to cure or prevent disease progression [11,160]. Regarding cancer GT, two modalities—namely corrective and death-induced therapies— were developed. Corrective GT aims to restore normal cell functions by either activating tumor suppressor genes or deactivating oncogenes, whereas death-induced GT triggers the death of cancer cells by activating different signaling pathways [161]. 

The success of GT relies on safe, effective, controllable, and targeted gene delivery, which is facilitated by using carriers or vectors able to effectively load genes through electrostatic interactions, travel throughout the circulatory system, protect genes from enzymatic degradation, accumulate within the tumor site, be effectively internalized within cancer cells, escape from endosomes after cellular uptake, and efficiently release the delivered cargo [9,125,127]. Gene delivery systems can be roughly classified as viral (e.g., adenoviral, AAV-adeno-associated virus, lentiviral) and non-viral vectors [11,162]. On the basis of the employed material, non-viral vectors are divided into lipidic, peptide-based, and polymeric nanocarriers [11]. Among all, polymeric nanoplatforms are considered ideal in terms of safety, biodegradability, biocompatibility, target-specific delivery, transfection efficiency, and cost-effectiveness. Such GT nanocarriers include chitosan, poly(lactic-co-glycolic) acid (PLGA), cyclodextrins, dendrimers, etc. [11]. Owing to its polymeric nature, PDA was recently employed in several studies as a potential gene carrier in GT.

A study in this regard is the one conducted by Zhang et al. who prepared polyethylenimine (PEI)-modified polydopamine (PDA)-based nanoparticles (PPNPs) for enhanced gene delivery under NIR illumination, considering the distinctive biocompatibility, photothermal conversion ability, and easy surface modification of PDA. The obtained PPNPs/DNA complexes exhibited a higher gene transfection ability than Lipofectamine 2000 (a commonly used transfection reagent), which tripled following NIR exposure. Furthermore, the modification of PEI using PDA led to a lower cytotoxicity in HepG2 cells and a reduced hemolytic effect [127]. Moving one step forward, the same research team modified PDA-NPs with low-molecular weight PEI and polyethylene glycol-phenylboronic acid (PEG-PBA) to design a pH-responsive platform for gene delivery (PDANP-PEI-rPEG). PDANP-PEI-rPEG/DNA complexes were stable in physiological pH conditions (pH = 7.4) and displayed an enhanced gene transfection level compared to a non-responsive carrier, as well as a quick endosomal escape following NIR irradiation. The local heat induced by NIR light promoted gene transfection and was attributed to the photothermal conversion property of PDA [125]. Both studies proposed that PDA could provide a synergistic effect between GT and PTT in cancer therapy.

##### Cancer Detection and Bio-Imaging

Cancer detection at early stages of development is the key to successful treatment, enabling a decrease in mortality incidence. Hence, the design of new, rapid and simple techniques for sensitive cancer cell detection would have a great impact on modern cancer management [163]. Although not yet deployed in the clinical diagnosis of cancer, nanotechnology has been extensively investigated as a promising tool for cancer detection and in vivo imaging. The peculiar features (i.e., optical, magnetic, and chemical) possessed by nanoprobes render the creation of imaging platforms that provide better contrast enhancement, multi-functional and multi-modal imaging, increased sensitivity, and controlled biodistribution—properties that could be translated into clinical advantages such as early disease detection, real-time assessment of disease progression, and personalized medicine [164]. The applicability of NPs in cancer detection involves the capture of tumor cells, biomarkers, DNA, proteins, or exosomes. A proper nanosized platform should exhibit a long circulation time, accumulate specifically within tumor tissues, and exert low toxicity to surrounding healthy tissues [165]. PDA was recently incorporated in several nanoplatforms for cancer detection. For instance, Ji et al. proposed Au-polydopamine functionalized carbon encapsulated Fe_3_O_4_ magnetic nanocomposites (Au/PDA/Fe_3_O_4_@C@PGC) for ultrasensitive detection of carcinoma-embryonic antigen, a useful biomarker for colorectal adenocarcinoma. In this case, PDA was used as both the reductant and template for one-step synthesis of gold NPs [166]. In another study, Wang et al. presented a highly sequence-specific gold@polydopamine-based (AuNP@PDA-hpDNA) nanoprobe for in vitro long-term detection of miRNA. This platform enabled continuous monitoring of the oncogenic miR-155 levels within MDA-MB-231 and HeLa cancer cells for up to 5 days, following a single administration [167].

Among all early diagnostic tools for cancer imaging, the most notable are magnetic resonance imaging (MRI), positron emission tomography (PET), ultrasound, and computed tomography (CT) [168]. Owing to their engineerable nature, NPs have been recognized as a new opportunity for maximizing the accuracy of cancer diagnostics [169]. Furthermore, NPs offer greater biocompatibility and reduced toxicity in comparison to conventional contrast agents. NPs that are currently under development for cancer imaging include gold NPs (applied in X-rays), magnetic NPs (applied in MRI), and hybrid NPs carrying iron oxide and gold in a polymer coating (applied in both CT and MRI) [170]. PDA was also integrated into nanoplatforms designed for cancer imaging. One such example is the study conducted by Dong et al., who developed PDA-based NPs loaded with indocyanine green (ICG), doxorubicin (DOX), and manganese ions [PDA-ICG-PEG/DOX(Mn)] that permit imaging-guided chemotherapy and PTT. The authors showed that the NPs are able to confer contrast under T1-weighted MRI. The MRI-guided chemo-photothermal therapy provided a prominent synergistic anticancer effect compared to the respective single treatment modalities in 4T1 tumor-bearing female mice [171].

##### Nanotheranostics

Theranostics, which is in essence a portmanteau of therapy and diagnostic, has emerged as a targeted, safe, and efficient approach to the development of more specific and individualized therapies in the treatment of various diseases [172]. With the emergence of nanotechnology and nanomaterials, the concept of nanotheranostics was introduced [173] and became a fast-growing pharmaceutical field, enabling the simultaneous drug delivery and monitoring of drug distribution, drug release, and therapeutic efficiency through a single nanoscale platform. Numerous nanoparticle-based theranostic agents have been exploited so far, including liposomes, dendrimers, micelles, carbon nanotubes, magnetic nanoparticles, gold nanoparticles, silica, and polymeric nanoparticles [172,174]. Melanin and melanin-like nanomaterials were reported as effective theranostic agents. For instance, Mao et al. constructed PDA-PEG NPs loaded with fluorescein isothiocyanate (FITC)-labeled hairpin DNA (hpDNA) and doxorubicin as a theranostic nanoprobe to monitor the expression of miRNAs and facilitate the early diagnosis and treatment of cancer. The authors demonstrated that the obtained nanoplatform enabled the real-time detection of specific miRNAs and allowed cancer therapy in living cells and mice [130]. Shi and colleagues fabricated hollow mesoporous silica NPs loaded with ultrasmall Fe_3_O_4_ NPs (USIONPs) and coated with PDA (finally abbreviated HMS-Fe_3_O_4_@PD) as a multifunctional platform in cancer theranostics. In this study, PDA served as a stabilizer and NIR light absorber in PA imaging and PTT, while USIONPs were selected for T1-weighted MRI. HMS-Fe_3_O_4_@PD showed hemocompatibility, exerted a potent photothermal ablation effect on 4T1 breast cancer cells, inhibited tumor growth in xenograft-bearing mice under laser irradiation, completely suppressed the tumor growth following both PTT and RT, and increased MRI and photoacoustic imaging signal overtime [175].

#### 4.2.3. Biosafety and Metabolism of MEL-NPs

The antitumor efficiency and wide applicability of melanin-functionalized nanoparticles have been demonstrated in numerous studies, as shown in Section 4.2.2. However, if they are to be part of the next generation of cancer treatment, concerns regarding their biocompatibility and safety should be addressed. Thereafter, gathering as much information as possible in order to portrait a complete toxicological profile of MEL/PDA-NPs will be essential.

First and foremost, in contrast to other nano-systems that have been developed so far (i.e., liposomes, dendrimers, micelles, polymeric, and inorganic nanoparticles), melanin has the advantage of being already produced within human melanocytes as nanosized particles [136,176]. The inherent biocompatibility has been described as one of the main advantages of natural melanin, and fortunately, it is retained by its bio-inspired synthetic copy known as PDA [97]. A recent study on human epidermal keratinocytes (HEKa) reported that PDA-NPs act like artificial melanosomes by mimicking their intracellular behavior in terms of trafficking and distribution and accumulating preferentially in the perinuclear area of the cells without causing significant cytotoxicity (cell viability of ~90% following a 3 day treatment) [144]. Another in vitro study indicated a selective cytotoxicity of PDA-functionalized nanoparticles and cellular uptake within cancer cells (i.e., human cervical carcinoma cells (HeLa) and human breast adenocarcinoma cells (MCF-7)) compared to healthy cells (i.e., human embryonic lung fibroblasts (HELF) and human normal liver cells (L02)) [119].

Regarding in vivo biosafety, Zhang et al. showed that melanin-based nanoliposomes (Lip-Mel) caused no significant side effects in treated mice and undetectable toxicity to primary organs highlighted by negligible variations of liver, kidney, and heart functional markers. Likewise, Chu et al. demonstrated that black sesame melanin nanoparticles encapsulated in liposomes exerted no long-term in vivo toxicity or negative impact on the liver and kidney functions of mice following subcutaneous administration [177]. Moreover, PDA can be strategically employed as a functional layer able to reduce the toxicity of other nanosystems. For instance, PDA coating was shown to attenuate the in vivo blood immunogenicity of uncoated quantum dots and cadmium selenide, as well as to reduce the tissue inflammation caused by poly(l-lactic acid) [178].

The toxicity of nanoparticles is also related to their pharmacokinetic properties (i.e., absorption, distribution, metabolism, and excretion profiles) [179]. The primary administration route for MEL/PDA-NPs used during in vivo studies was parenteral (i.e., subcutaneous or intravenous) [107,177,180]. Liu et al. conducted a complex biodistribution study on 68Ga-labeled pH-sensitive MEL-NPs in H22 tumor-bearing BALB/c mice, revealing their preferential uptake in the liver, clearance through the hepatobiliary system, and retention within the tumor site [180]. In vivo trafficking studies also showed the ability of melanin-functionalized NPs to accumulate and prolong antigen retention within the lymph nodes following subcutaneous injection [121,123].

All in all, preclinical studies revealed promising results in terms of safety and biocompatibility of melanin and PDA-based NPs, which might be attributed mainly to their close resemblance to the natural pigment. 

## 5. Concluding Remarks and Future Perspectives

The last few decades were marked by notable findings concerning melanin, the natural biopolymer with an impressive collection of unique physicochemical and biological features, converting this molecule into a promising candidate with high impact in nanotechnology and implicitly in biomedicine. The biological properties of natural melanins, as antioxidant, photoprotective, anti-inflammatory, and mainly anticancer effects, were intensively studied and explicitly described in this review; still, some aspects need further research to define the anticancer mechanism of action, which is not fully elucidated at present yet might lead to remarkable results in anticancer therapy.

Its exclusive intrinsic physicochemical properties, such as broadband light absorption in VIS, UV and NIR, the capacity to convert photon energy into heat, radical-scavenging properties, paramagnetism, semi-conductivity, chelation ability for organic and inorganic compounds, and biocompatibility, have propelled melanin into the field of nanotechnology, as demonstrated by the considerable number of melanin-like nanoparticles that have been designed in recent years, highlighting the outstanding potential of melanin in oncology as a feasible approach in chemotherapy, phototherapy and photodynamic therapy, radiopharmaceutical therapy, immunotherapy, gene therapy, cancer detection and bio-imaging, and nanotheranostics. Despite the promising preclinical research on melanin-like nanoparticles, the data remain insufficient on the exact composition of these nanoplatforms [99], the metabolism and biodegradation of each type of melanin nanoparticle, and the impact on organisms from long-term administration. Further studies should focus on these aspects, which could provide a bridge towards the translation from preclinical to clinical data. 

To the best of our knowledge, to date there has been no ongoing clinical trial evaluating the pharmaco-toxicological profile of melanin-functionalized nanoparticles in cancer therapy. However, current preclinical results displaying the favorable antitumor activity and biocompatibility of MEL-NPs might speed up their clinical implementation. Among all promising areas of cancer treatment involving melanin-based nanotechnology that were presented in this article, chemotherapy seems to be the most probable direction to be clinically explored in the near future, considering its wide application as first-line cancer treatment method and the plethora of studies conducted thus far on melanin nanoparticles as drug carriers, especially for some chemotherapeutic agents that are already approved for clinical practice (e.g., doxorubicin and paclitaxel).

As future perspectives in terms of melanin potential in oncology, the application of melanin has been proposed as a ”molecular target” for targeted anticancer therapy [89,90,91], or as a diagnostic tool together with Raman spectroscopy and multivariate data analysis for the diagnosis of dysplastic nevi [181]. Another direction that should be explored is the use of melanin as a carrier for natural compounds with anticancer activity that present a hydrophobic character and reduced bioavailability.

## Figures and Tables

**Table 1:** Relevant studies highlighting the therapeutic properties exerted by melanins from different sources.

Source	Effect	Observations	Tested Concentrations	Reference
Fungal melanin from *Aspergillus nidulans*	Antioxidant	inhibition of 5-thio-2-nitrobenzoic acid (TNB) oxidation; scavenging activity on the tested oxidants (i.e., H_2_O_2_ and HOCl)	25, 50, and 100 µg/mL (in vitro)	[46]
Melanin from the muscles of Gallus domesticus Brisson	Antioxidant	concentration-dependent scavenging of DPPH and superoxide radicals; inhibition of lipid peroxidation	20–3000 µg/mL (in vitro)	[47]
Bacterial melanin from *Streptomyces glaucescens* NEAE-H	Antioxidant Anticancer Anti-hemolytic	scavenging and neutralization of ABTS radical; ↑ mortality and cytotoxicity in HFB4 skin cancer cells; neutralization of free radicals and protection of erythrocytes from membrane destruction/lysis	1.56–100 µg/mL; IC_50_ = 16.34 ± 1.31 µg/mL (in vitro)	[48]
Bacterial melanin from *Pseudomonas maltophilia* AT18	Photoprotective	↑ viability of normal fibroblasts (NL-FB) post-UVA irradiation; inhibition of UVA-induced apoptosis; suppression of intracellular ROS generated by UVA	25–800 µg/mL (in vitro)	[49]
Bacterial melanin from *Pseudomonas otitidis* DDB2	Photoprotective	protection of NIH 3T3 mouse fibroblasts against UVB radiation; scavenging of ROS generated upon UVB irradiation	15.625–500 µg/mL (in vitro)	[50]
Herbal melanin from *Nigella sativa* seed coats	Immunomodulatory	↑ TNF-α, IL-6, and VEGF mRNA expression in human monocytic THP-1 cells and peripheral blood mononuclear cells (PBMC)	50 and 100 µg/mL (in vitro)	[51]
	Immunomodulatory	↑ IL-8 expression and production in human monocytic THP-1 cells and peripheral blood mono- nuclear cells (PBMC)	5–50 µg/mL (in vitro)	[52]
	Anticancer	↓ cell viability; ↑ generation of cellular ROS; apoptosis induction; ↓ Bcl-2 expression; ↑ Bad expression; ↑ cytochrome c expression; activation of caspase-3 and -7; ↑ JNK, cJun and ATF2 phosphorylation; ↓ ERK phosphorylation in HT-29 and SW620 colorectal adenocarcinoma cells	5–200 µg/mL (in vitro)	[53]
	Anticancer	↓ cell viability; cell growth arrest in G0/G1 and G2 phases; ↑ TLR4 protein expression; apoptosis induction in human acute monocytic leukemia THP-1 and human embryonic kidney HEK293 cells	7.8–500 µg/mL (in vitro)	[54]
B16F10 melanoma tumor lysates containing melanin (microneedle patch)	Anticancer	Melanin-mediated heat generation; promotion of tumor-antigen uptake by dendritic cells; ↑ antitumor vaccination against B16F10 tumors; complete tumor remission in BRAF^V600E^-mutated BP melanoma- and 4T1 breast carcinoma-bearing mice	around 50 µg of melanin/patch (in vivo)	[55]
Synthetic melanin	Immunomodulatory	↑ CD8+ T-cell responses and inhibition of tumor growth in BALB/c mice; ↑ efficiency of melanin as adjuvant in anticancer vaccines	0.5 μg of melanin bound to the gp100 epitope (gp100-melanin) (in vivo)	[56]

↑ increase; ↓ decrease; HOCl-Hypochlorous acid; DPPH—2,2-diphenyl-1-picrylhydrazyl; ABTS—2, 2′-azino-bis (3-ethylbenzothiazoline-6-sulfonic acid); TNF-α—tumor necrosis factor alpha; IL-6—Interleukin 6; VEGF—vascular endothelial growth factor; IL-8—Interleukin 8; Bcl-2—B-cell lymphoma 2; JNK—c-Jun N-terminal kinase; ATF2—Activating transcription factor 2; ERK—extracellular signal-regulated kinase; TLR4—toll-like receptor 4.

**Table 2:** Relevant studies highlighting the applications of melanin-based nanosystems in cancer treatment.

Application	Melanin Nanoplatform Type	Cancer Type	Observations	Concentration/Dosage	Reference
Chemotherapy	Doxorubicin-loaded MEL-NPs	Thyroid cancer	Chelating of doxorubicin (DOX) through π-π stacking and hydrogen bonding; ↓ viability of HTh74 and HTh74R thyroid cancer cells; ↑ therapeutic efficacy and cell internalization compared to free doxorubicin.	10, 20, 40, 80, 160 mg/L (in vitro)	[98]
	PDA-coated and alendronate-grafted paclitaxel (PTX) nanoparticles	Osteosarcoma	Targeted cancer treatment; sustained drug release; ↑ cytotoxicity against K7M2 wt osteosarcoma cells; ↑ accumulation in tumor, and ↓ side effects of PTX in K7M2 wt tumor-bearing mice	1, 5, 10, 50, 100 µg/mL (in vitro) 8 mg/kg equivalent concentrations of PTX (in vivo)	[99]
	PTX-loaded trastuzumab-decorated PDA-NPs (PDA NPs•Tmab@PTX)	Breast cancer	↓ viability of BT474, SKBR3, and HS5 cells HER2+ breast cancer cells; ↑ number of early apoptotic HER2+ breast cancer cells BT474; disintegration and ↓ viability BT474 spheroids	0.035, and 0.042 mg/mL (2D in vitro model) 0.035 mg/mL (3D in vitro model)	[100]
	RGD-modified polydopamine-paclitaxel-loaded poly (3-hydroxybutyrate-co-3-hydroxyvalerate) nanoparticles	Hepatocellular carcinoma	↓ cytotoxicity against L02 of PTX-free NPs; ↓ viability of HepG2 and SMMC-7721 cells; ↑ inhibitory effect on HepG2 and SMMC-7721 cell proliferation compared to free PTX; ↑ cellular uptake in HepG2 cells; ↑ PTX release at pH values of 5.0–6.5; ↓ tumor volume and weight in HepG2 tumor-bearing mice	0.1, 0.5, 1, 2.5, 5, 10 µg/mL (in vitro) 4 mg/kg (in vivo)	[101]
	Doxorubicin-loaded polyethylene glycol functionalized MEL-NPs	Breast cancer	Sustained and extended release of doxorubicin; ↓ proliferation of MDA-MB-231 breast cancer cells;	0.125, 0.250, 0.500 mg (in vitro)	[102]
	Curcumin-loaded silver-decorated melanin-like polydopamine/mesoporous silica composites	Cervical and Taxol-resistant non-small cell lung cancers	↓ hemolytic activity and biocompatibility; pH- and ROS-responsive release of curcumin; prolonged inhibition of Escherichia coli and Staphylococcus aureus bacterial growth; ↑ chemotherapeutic efficiency against HeLa (human cervical) and A549/TAX (Taxol-resistant non-small cell lung) cancer cells compared to free curcumin.	≤ 500 µg/mL (in vitro)	[103]
	Gambogenic acid-loaded functional polydopamine nanoparticles (GNA@PDA-FA SA NPs)	Breast cancer	↓ 4T1 (breast cancer cells) cell viability; ↓ IC_50_ value compared to raw GNA; ↑ targeting effect of GNA against 4T1 cells; inhibition of tumor growth in 4T1 xenograft-bearing BALB/C mice	0.78–310 µM (in vitro) 24 mg/kg (in vivo)	[104]
	Iron-chelated doxorubicin-loaded folic acid-conjugated polyethylene glycol (PEG)-coated polydopamine nanoparticles (DOX@Fe-PDA/FA-PEG NPs)	Breast cancer	↑ pH responsiveness of the PDA-modified NPs and pH-dependent release of DOX; ↑ DOX release under acidic conditions; sustained DOX release; ↑ cell uptake compared to free DOX; ↓ MCF7 (breast cancer cells) cell viability; ↑ intracellular ROS in MCF7 cells	0.1093–3.5 µg/mL (in vitro)	[105]
	Doxorubicin-loaded polyethylene glycol-modified polydopamine nanoparticles (PDA-PEG-DOX)	Breast cancer	↓ MDA-MB-231 (breast cancer cells) cell viability; ↓ pro-caspase 3 expression level; accumulation within the MDA-MB-231 cell nucleus and lysosomes; ↓ mitochondrial membrane potential	0.5, 1, and 5 µg/mL (in vitro)	[106]
	Doxorubicin-loaded triphenylphosphonium- functionalized polyethylene glycol-modified polydopamine nanoparticles (PDA-PEG-TPP-DOX)	Breast cancer	↓ MDA-MB-231 cell viability; ↓ pro-caspase 3 expression level; ↓ mitochondrial membrane potential; mitochondria-targeted anticancer effect; ↓ long-term drug resistance.	0.5, 1, and 5 µg/mL (in vitro)	[106]
Radio (pharmaceutical) Therapy	Melanin-covered silica nanoparticles (MNs)	-	↓ hematologic toxicity in mice exposed to external gamma radiation and radioimmunotherapy	50 mg/kg (in vivo)	[107]
	Melanin nanoparticles (MNPs)	-	↓ gamma radiation-induced cytotoxicity in Chinese hamster ovary cells	6.25, 12.5, 25 and 50 µg/mL (in vitro)	[108]
	^131^I-labeled PEGylated polydopamine nanoparticles loaded with sanguinarine and metformin (^131^I-PDA- PEG-SAN-MET)	Breast cancer	↓ 4T1 cell viability; induction of 4T1 cell apoptosis; relieved tumor hypoxia in 4T1 tumor-bearing nude mice.	NPs containing 4 mg/kg of SAN, 8 mg/kg of MET, 200 mCi of ^131^I (in vivo)	[109]
	PEGylated polydopamine nanoparticles loaded with ^131^I and DOX (^131^I-PDA-PEG/DOX)	Breast cancer	↓ 4T1 cell viability; ↑ cellular ^131^I uptake delivered by PDA-PEG; inhibited tumor growth, reduced tumor size, and prolonged survival rate in BALB/c mice bearing 4T1 xenografts.	10 mg/kg of PDA-PEG, 20 mCi of ^131^I (in vivo)	[110]
Phototherapy	Arginine-doped synthetic melanin nanoparticles (SMNPs)	Breast cancer	↑ photothermal efficiency following arginine introduction within the PDA structures of SMNPs; ↓ 4T1 cell viability; ↓ tumor volume and weight in 4T1 xenograft-bearing female BALB/c nude mice.	12.5, 25, 50, 100, 200 μg/mL (in vitro) 10.0 and 20.0 mg/kg (in vivo)	[111]
	RGD- and beclin 1-modified and PEGylated MEL-NPs	Anticancer	Induced autophagy and cytotoxicity; ↓ cell viability upon NIR irradiation in cancer cells; tumor regression in BALB/c nude mice at 43 °C	50 μg/mL (in vitro) 34 mg/kg (in vivo)	[112]
	Cisplatin prodrug Pt (IV) MEL-NPs	Prostate cancer	↓ viability of PC3, DU145, and LNCaP prostate cancer cells; induction of mitochondrial membrane depolarization in PC3 cells; ↑ cell uptake ability; synergistic photothermal therapy and chemotherapy properties; potent photothermal conversion efficiency (29.6%); biocompatibility; prolonged the blood circulation time and ↓ tumor growth in BALB/c mice.	10, 20, 30 µM (in vitro) 200 µL (in vivo)	[113]
	Gemcitabine-loaded dual-functional melanin-based nanoliposomes	Pancreatic cancer	Synergistic antitumor effect between melanin and gemcitabine; ↑ therapeutic efficiency; potent conversion of NIR light into thermal energy in the presence of MEL; photothermal conversion efficiency of MEL uninfluenced by liposomal encapsulation and drug loading; ↓ cell viability of BxPC-3 pancreatic cancer cells; controlled and enhanced drug release to the tumor sites via hyperthermia; no systemic toxicity to BxPC-3 tumor-bearing mice	50 mg/kg (in vivo)	[114]
	Docetaxel-loaded polydopamine-functionalized CA-(PCL-ran-PLA) nanoparticles	Breast cancer	↑ drug loading content, and encapsulation efficiency; effective target delivery of drugs to tumor sites by incorporating AS1411 aptamers; synergistic chemo-photothermal effect; ↓ proliferation of MCF-7 breast cancer cells; ↑ survival time, and ↓ side effects in mice; ↓ tumor volume in vivo	0.25–25 μg/mL (in vitro) 10 mg/kg (in vivo)	[115]
	PDA/transferrin hybrid NPs	Malignant melanoma	↑ apoptosis when associated with UV irradiation in B16F10 mouse melanoma cells, J774A.1 mouse macrophages, and in an organotypic melanoma spheroid model; lack of cytotoxicity or proliferation impairment of PDA-NPs in B16F10 and J774A.1;	5–160 µg/mL (in vitro)	[116]
	Hyaluronic acid-decorated polydopamine nanoparticles with conjugated chlorin e6 (HA–PDA–Ce6)	Colorectal carcinoma	↓ dark toxicity; ↑ photodynamic and photothermal activities upon laser illumination; ↑ uptake and penetration in vitro and in vivo; ↑ cytotoxicity and apoptosis in HCT-116 cells following the combined laser treatment; inhibited tumor growth in HCT-116 tumor-bearing mice.	IC_50_ = 33.07 ± 12.92 μg/mL (in vitro) 0.65 mg/kg (in vivo)	[117]
	Epirubicin-hybrid polydopamine nanoparticles (E/PCF-NPs)	Breast cancer	pH sensitive drug release; ↑ cytotoxicity against 4T1 cells; inhibited survival rate and induced cell apoptosis 4T1 cells; ↑ ROS generation; ↓ NAD+/NADH; complete tumor regression in 4T1 tumor-bearing mice	IC_50_ = 1.3 ± 0.2 μg/mL (in vitro) 5 mg/kg drug dose (in vivo)	[118]
	Folate-modified PDA nanoparticles loaded with a cationic phthalocyanine-type photosensitizer (PDA-FA-Pc)	Breast cancer Cervical cancer	Non-measurable toxicity of PDA-FA-Pc without illumination; ↓ dose-dependent survival rate of MCF-7, HeLa, HELF, and L02 cells following illumination; ↑ cytotoxicity against tumor cells (MCF-7, HeLa) compared to healthy cells (HELF, L02); ↓ tumor volume and weight in MCF-7 and HeLa xenograft-bearing female Kunming mice.	0.15, 0.3, 0.6, 1.2 and 2.4 mg/mL (in vitro) 43.5 mg/kg (in vivo)	[119]
	Chlorin e6-conjugated PDA nanospheres	Hepatocellular carcinoma	Simultaneous PTT and PDT therapy; ↑ internalization within HepG2 cells; ↓ cell viability of HepG2 cells; tumor regression in HepG2 tumor-bearing male BALB/c-nude mice	Ce6 concentration 0.1–8 μg/mL (in vitro) 20 μg/mL PDA and 5 μg/mL Ce6 (in vivo)	[120]
	PDA nanoparticles carrying tumor cell lysate (TLC) (TCL@PDA NPs)		Delayed cancer progression in tumor-bearing mice; ↑ antigen uptake, BMDCs (bone-marrow-derived dendritic cells) maturation, and Th1-related cytokines secretion; ↑ CD4+ and CD8+ T cells; delayed tumor development by empty PDA-NPs	300 μg TLC	[121]
Immunotherapy	Polydopamine-coated mesoporous silica nanoparticles containing thiolated ovalbumin and ammonium bicarbonate (MSNs-ABC@PDA-OVA)	Malignant melanoma	Rapid antigen release and endosome escape under laser illumination; ↑ activation and maturation of dendritic cells; antigen specific CD8+ and Th1 CD4+ T cell responses; melanoma eradication with a cure rate of 75%; strong immunological memory; inhibition of tumor recurrence and metastasis in C57BL/6 mice.	25 µg OVA/mouse (in vivo)	[122]
	Antigen-ovalbumin-loaded polydopamine nanoparticles (OVA@Pdop-NPs)	Colon cancer	Lack of cytotoxicity and ↑ cellular uptake in bone marrow-derived dendritic cells (BMDCs); ↑ maturation of dendritic cells; ↑ expression of major histocompatibility complex, costimulatory molecules, and cytokines; activation of OVA-specific cytotoxic CD8+ T cells; ↑ production of memory CD4+ and CD8+ T cells; ↓ tumor growth in OVA-MC38 colon tumor-bearing mice	0.5–100 μg/mL (in vitro) 100 μg/mice OVA content (in vivo)	[123]
	Natural melanin nanoparticles coated with cancer cell membrane (M@C NPs)	Breast cancer	↑ antitumor activity; ↑ levels of CD8+ T cells and cytokines; ↑ 4T1 cell cytotoxicity and ↓ cell invasion under laser radiation; ↑ expression of calreticulin proteins under irradiation suggesting immunogenic cell death of 4T1 cells; ↑ tumor targeting ability, ↓ levels of IL- 12 and IL-6, and synergistic effect with immunoblocking inhibitors (IDOi) leading to ↓ tumor volume and growth in mice.	≤ 1000 μg/mL (in vitro)	[124]
Gene Therapy	pH-responsive polydopamine nanoparticles modified with polyethylenimine and polyethylene glycol-phenylboronic acid (PDANP-PEI-rPEG)	Hepatocellular carcinoma (in vitro) Malignant melanoma (in vivo)	Stability to physiological pH (7.4); ↑ gene transfection levels; ↑ photothermal conversion ability; quick endosomal escape;	0.4–1.5 mass ratio PDANP to DNA (in vitro) 50 μL (in vivo)	[125]
	DNA-polydopamine-MnO_2_ nanocomplex (DP-PM)	Breast cancer	↓ viability of MCF7 cells; ↓ tumor volume and weight in MCF7 xenograft-bearing BALB/c nude mice; glutathione-triggered release of Mn^2+^ to activate intracytoplasmic DNAzyme ↑ Egr-1 mRNA cleavage activity of DNAzyme and ↓ of Egr-1 protein in tumor cells; synergistic tumor ablation upon NIR irradiation.	5–50 μg/mL (in vitro)	[126]
	Polyethylenimine-modified polydopamine nanoparticles (PPNPs)	Hepatocellular carcinoma	↓ cytotoxicity to HepG2 cells; ↑ gene transfection levels compared to Lipofectamine 2000 at mass ratios of 23 and 30; tripled gene transfection levels following NIR irradiation; lack of hemolytic effect.	10–30 mass ratio PPNPs to DNA (in vitro)	[127]
Cancer Detection and Bio-Imaging	Mesoporous polydopamine carrying sorafenib and SPIO nanoparticles (SRF@MPDA-SPIO NPs)	Hepatocellular carcinoma	↑ MRI contrast; ↑ R2 (1/T2) values; MRI-guided ferroptosis; responsive release of ferric ions and sorafenib to stimuli (pH, temperature); effectively conversion ability of NIR light; reduced tumor volume and weight in HCT-116 tumor-bearing mice	100 μL (in vivo)	[128]
	Ions (Fe^3+^, Bi^3+^, I^+^)-doped melanin nanoparticles conjugated with EGFR antibody (iMNPs)	Hepatocellular carcinoma	↑ contrast intensity in T1-w MRI and CT; specific targeting of EGFR-overexpressed HepG2 cells observed by MRI and CT imaging; ↑ contrast of MRI/CT/SPECT images in xenograft-bearing-mice	200 μL (in vivo)	[129]
Nanotheranostics	PDA-based theranostic nanoprobe loaded with fluorescein isothiocyanate (FITC)-labeled hairpin DNA (hpDNA) and doxorubicin	Breast cancer	↓ viability of 4T1 breast cancer cells; real-time detection of the dynamic expression of specific miRNAs; ↓ tumor volume in 4T1 xenograft-bearing male BALB/c-nu mice	2.5, 5, 10, 20 μg/mL of doxorubicin (in vitro)	[130]
	Cu (II)-doped polydopamine-coated gold nanorods	Squamous cell carcinoma	↑ physiological stability, biocompatibility, photothermal performance, and blood circulation time; computer tomography imaging and magnetic resonance imaging functions; ↓ tumor volume and weight; lack of short-term toxicity against liver and renal functions in BALB/c mice	25–500 µg/mL (in vitro) 50 μL of 5 mg/mL (in vivo)	[131]
	Mn^2+^ -coordinated PDA-modified doxorubicin-loaded poly (lactic- co-glycolic acid) (PLGA) NPs	Colon cancer	↑ permeability and retention; ↑ ability of NIR photothermal transduction in vitro and in vivo; chemo-photothermal synergistic effect; ↑ DOX release; ↓ viability of CT26 murine colorectal carcinoma cells; stronger efficacy in killing cancer cells under NIR irradiation; efficient cellular uptake; ↓ tumor growth in CT26 tumor-bearing mice; no acute side effects in vivo	≤ 200 µg/mL (in vitro) 20 mg/kg (in vivo)	[132]

↑ increase, ↓ decrease; RGD—arginine-glycine-aspartic acid peptide; HELF—human lung fibroblasts; L02—human liver cell line.
